# Use of Modified *Clostridium perfringens* Enterotoxin Fragments for Claudin Targeting in Liver and Skin Cells

**DOI:** 10.3390/ijms20194774

**Published:** 2019-09-26

**Authors:** Laura-Sophie Beier, Jan Rossa, Stephen Woodhouse, Sophia Bergmann, Holger B. Kramer, Jonas Protze, Miriam Eichner, Anna Piontek, Sabine Vidal-y-Sy, Johanna M. Brandner, Gerd Krause, Nicole Zitzmann, Jörg Piontek

**Affiliations:** 1 Charité-Universitätsmedizin Berlin, Institute of Clinical Physiology, Hindenburgdamm 30, 12203 Berlin, Germany; Laura-Sophie.Beier@charite.de (L.-S.B.); miriam.eichner@googlemail.com (M.E.); 2 Leibniz-Forschungsinstitut für Molekulare Pharmakologie (FMP), Robert-Rössle-Straße 10, 13125 Berlin, Germany; JanRossa@gmx.de (J.R.); Protze@fmp-berlin.de (J.P.); dr.anna.piontek.ext@bayer.com (A.P.); GKrause@fmp-berlin.de (G.K.); 3 Oxford Glycobiology Institute, Department of Biochemistry, University of Oxford, South Parks Road, Oxford OX1 3QU, UK; Stephen.woodhouse@cardiov.ox.ac.uk (S.W.); nicole.zitzmann@bioch.ox.ac.uk (N.Z.); 4 Department of Dermatology and Venerology, University Hospital Hamburg Eppendorf, 20246 Hamburg, Germany; s.bergmann@t-online.de (S.B.); s.vidal-y-sy@uke.de (S.V.-y.-S.); brandner@uke.de (J.M.B.); 5 Oxford Centre for Gene Function, Department of Physiology, Anatomy and Genetics, University of Oxford, Oxford OX3 7DQ, UK; h.kramer@lms.mrc.ac.uk

**Keywords:** *Clostridium perfringens* enterotoxin, claudin-1, Hepatitis C Virus, viral entry, epidermal barrier, reconstructed human epidermis, claudin targeting

## Abstract

Claudins regulate paracellular permeability in different tissues. The claudin-binding domain of *Clostridium perfringens* enterotoxin (cCPE) is a known modulator of a claudin subset. However, it does not efficiently bind to claudin-1 (Cldn1). Cldn1 is a pharmacological target since it is (i) an essential co-receptor for hepatitis C virus (HCV) infections and (ii) a key element of the epidermal barrier limiting drug delivery. In this study, we investigated the potential of a Cldn1-binding cCPE mutant (i) to inhibit HCV entry into hepatocytes and (ii) to open the epidermal barrier. Inhibition of HCV infection by blocking of Cldn1 with cCPE variants was analyzed in the Huh7.5 hepatoma cell line. A model of reconstructed human epidermis was used to investigate modulation of the epidermal barrier by cCPE variants. In contrast to cCPEwt, the Cldn1-binding cCPE-S305P/S307R/S313H inhibited infection of Huh7.5 cells with HCV in a dose-dependent manner. In addition, TJ modulation by cCPE variant-mediated targeting of Cldn1 and Cldn4 opened the epidermal barrier in reconstructed human epidermis. cCPE variants are potent claudin modulators. They can be applied for mechanistic *in vitro* studies and might also be used as biologics for therapeutic claudin targeting including HCV treatment (host-targeting antivirals) and improvement of drug delivery.

## 1. Introduction

Claudins, tetraspan transmembrane proteins, are key structural and sealing components of tight junctions (TJ). In endo- and epithelia, they seal the paracellular space and regulate the paracellular ion permselectivity of the tissue and contribute to membrane polarity [1]. Besides their role in the apical TJ, claudins are also localized outside of the TJ complex, indicating that claudins might function apart from their classical role regulating paracellular permeability. Claudins have been shown to be important factors in cell signaling, cell adhesion, epithelial to mesenchymal transition, cell migration, mucosal homeostasis as well as disease pathogenesis (for review see [2]).

Various claudins serve as cell-surface receptors for pathogens, e.g., the *Clostridium perfringens* enterotoxin. The C-terminal domain of this toxin (cCPE) interacts with a subgroup of the claudin protein family (CPE-receptor claudins, e.g., claudin-3 (Cldn3), -4, -6, -9) [3]. cCPE was used in several studies to modulate the claudin-based barrier function of TJs [4]. To overcome the limitation of targeting CPE-receptor claudins only, claudin-subtype specificity of cCPE has been adapted by structure-guided mutagenesis and baculoviral display system. This enabled targeting of Cldn1-9 with the broad specificity binder cCPE-S305P/S307R/S313H (cCPE-SSS) [5,6], preferential binding to Cldn4 with cCPE-L254A/S256A/I258A/D284A [7] or preferential binding to Cldn5 with cCPE-Y306W/S313H and cCPE-N218Q/Y306W/S313H [6,8]. These variants bind with nanomolar affinities to their respective receptors [4,7,9,10]. The high affinity for their receptors as well as the claudin-specific binding renders cCPE variants appropriate tools for claudin targeting. The comparatively low production cost of the small recombinant protein and the concomitant targeting of a defined set of claudins with one molecule are convenient advantages over monoclonal antibodies against claudins.

Similar to other junctional proteins [11,12], claudins are employed as virus receptors. Cldn1 is an essential co-factor in hepatitis C virus (HCV) entry into hepatocytes [13]. Blocking of viral entry is a potent target for anti-HCV therapies with pan-genomic activity against multiple HCV subtypes; however, appropriate agents are not yet clinically available [14]. The multi-claudin binder cCPE-SSS might be a suitable agent to inhibit HCV receptor formation by binding Cldn1 and thereby blocking HCV entry into hepatocytes. HCV is a hepatotropic virus that has a high propensity to establish a persistent infection in human liver. HCV primarily infects human hepatocytes, which over time leads to chronic inflammation, progressive fibrosis, and development of hepatocellular carcinoma [15].

According to the World Health Organization, 3% of the world’s population is infected with HCV [16]. About 15 to 45% of infected people spontaneously clear the virus within six months. The remaining 60 to 80%, currently ~71 million people worldwide, develop a chronic HCV infection, which, in a significant number of cases, will lead to cirrhosis or liver cancer. Each year, around 400,000 people die from hepatitis C-related cirrhosis or hepatocellular carcinoma [17]. Thus, even with the high success-rates of new direct-acting antivirals, HCV remains a major burden on global health.

HCV entry into hepatocytes is the first step of the viral life cycle resulting in productive viral infection [13,18]. The entry pathway consists of three steps: (1) viral attachment to hepatocytes; (2) receptor-mediated endocytosis of viral particle; and (3) endosomal fusion [19]. Viral entry is mediated by the viral envelope glycoproteins E1 and E2 and several host entry factors. These include heparan sulfate, tetraspanin cluster of differentiation 81 (CD81), scavenger receptor class B type I (SR-BI), and the TJ proteins Cldn1 [20] and occludin [21,22]. Subsequent studies demonstrated that Cldn6 and -9, displaying high levels of homology with Cldn1, are also able to mediate HCV entry into non-permissive cell lines [23,24]. However, in hepatoma cells, Cldn6 and Cldn9 appeared to function weaker than Cldn1 [24]. In addition, low expression levels of Cldn6 and Cldn9 in human hepatocytes seem to impede their efficient use as HCV entry factors [25].

It was suggested that Cldn1 plays a dual role in HCV entry [25]: (i) formation of a HCV co-receptor complex with CD81 [26] and (ii) direct interaction with the virus envelope E1E2 proteins [27]. While predominantly localized at the TJ, Cldn1 also localizes on the basolateral membrane of hepatocytes. Notably, this pool of Cldn1 outside of the TJ co-localizes with CD81 and allows the formation of the CD81-Cldn1 co-receptor complex for HCV entry [26,28].

HCV entry is a major target of the host neutralizing response [15,19,29] and a target for antiviral immunopreventive and therapeutic strategies (for review see [13,19,30,31]). In particular, use of blocking anti-Cldn1 antibodies showed promising results in blocking viral cell entry and cell-to-cell transmission in vitro and in vivo [14,30,32]. Treatment with an anti-Cldn1 monoclonal antibody cured chronically infected human liver chimeric mice [33]. As an alternative HCV treatment, in this study, we investigated the potential of claudin-binding cCPE variants to block HCV infection.

Cldn1 is also one of the main sealing claudins in human epidermis [34,35], a stratified epithelium, where multiple layers of cells protect the body against water loss, ultraviolet radiation and penetration of harmful xenobiotics [36]. There are two physiologic barriers serving this function: dead keratinocytes containing lipids and proteins, i.e., the *stratum corneum* (SC), and the TJ between the viable cells of the *stratum granulosum* (SG) [36,37]. Claudin targeting by cCPE variants might modify the latter barrier [37,38,39]. Thus, we used a skin model in addition to the HCV-hepatoma model to test cCPE variants in two different Cdn1-dependent cellular processes.

We utilized models of reconstructed human epidermis (RHE), which recreate all epidermal layers including the TJ-containing SG and the biologically dead yet highly dynamic SC. In order to develop a functional SC, the apical side of the keratinocytes is exposed to air, creating an air-liquid interface (ALI) [40]. RHE enables the analysis of keratinocyte-specific responses within the epidermis, making it an ideal model for this study, in which the capability of the cCPE variants to open the epidermal barrier was tested.

In this study, we investigated the potential of cCPE variants to target processes that depend on extra-junctional or junctional claudins. Targeting of extra-junctional Cldn1 in hepatocytes, which serves as a co-receptor for HCV infection, by cCPE-SSS efficiently inhibited HCV infection. In RHE, on the other hand, we were able to modify the junctional epidermal barrier by targeting claudins in the SG. Thus, we showed that cCPE variants are suitable for directed claudin targeting.

## 2. Results

### 2.1. Structure Homology Modeling and FRET Analyses Suggest that cCPE-Cldn1 Interaction Interferes with Hepatitis C Virus Entry into Host Cells

**Figure 1:**
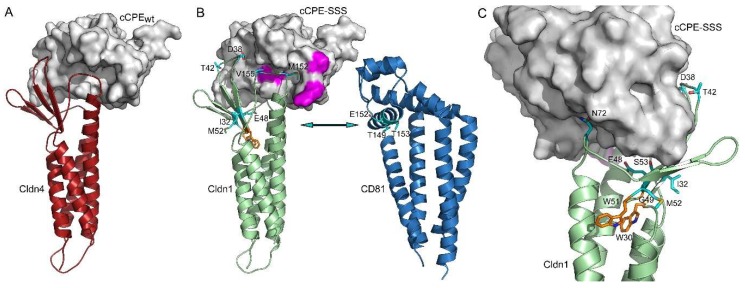
Homology modeling indicates that cCPE-Cldn1 interaction interferes with formation of HCV receptor complex. (**A**) X-ray structure (PDB ID: 5B2G, [43]) of Cldn4 (cartoon, red) interacting with cCPEwt (surface, gray) (**B**) Binding of cCPE-SSS to Cldn1 likely buries large parts of the extracellular domain of Cldn1 preventing Cldn1-CD81 interaction (arrow). Homology model of Cldn1 in complex with cCPE-SSS (template PDB ID: 5B2G) and CD81 (PDB ID: 5TCX). CD81 (blue) and Cldn1 (green) shown as cartoon; cCPE-SSS shown as the surface in gray and the substitutions (S305P, S307R, S313H) enhancing Cldn1 binding in magenta. Cldn1-CD81 interaction-relevant residues shown as cyan sticks (**C**) Other perspective highlighting Cldn1 residues involved in HCV entry [20,26] as cyan sticks. D38, T42, E48, S53, N72, M152, V155, reported to be involved in HCV infection are masked by cCPE. Involvement of W30, G49, W51 (sticks, orange) in HCV infection is likely to be due to a role in claudin folding [44]. I32 and M52 reported to be HCV relevant are not covered by cCPE.

HCV entry into hepatocytes depends on Cldn1 as a HCV co-receptor. We hypothesized that HCV infection might be inhibited by a Cldn1-binding cCPE variant since its interaction with Cldn1 on the cell surface might block the virus entry. The interaction mechanism of cCPE with defined claudin subtypes was well characterized by binding, mutagenesis and modeling studies. The motif (N/D(P-1) P(P) L/M/V(P+1) V/T(P+2) P/A/D(P+3)) in the extracellular segment 2 (ECS2) of claudins binds to the claudin-binding pocket of cCPE consisting of a triple tyrosine and a triple leucine pit [6,7,10,41]. This mechanism was confirmed by complex structures of cCPE bound to Cldn19 or Cldn4 [42,43]. Using these structures, a homology model of a complex between the Cldn1-binding cCPE-SSS and Cldn1 was generated (Figure 1). The model clearly indicates that binding of cCPE-SSS to the extracellular domains of Cldn1 buries most of the residues, which were shown to be involved in Cldn1-CD81 HCV receptor complex formation [20,26]. In particular, this is the case for D38, E48, T42, N72 in ECS1 and M 152 and V155 in ECS2. Other residues involved in Cldn1-CD81 association include W30, G49 and W51, which are part of the claudin consensus sequence and presumably involved in the general fold of claudins [44]. In summary, the modeling suggests that cCPE binding to Cldn1 interferes with formation of the HCV receptor/entry factor complex which includes Cldn1 and CD81. Thus, cCPE-SSS might block HCV entry.

Cldn1 and CD81 interact with each other in the plasma membrane outside of TJ. This interaction can be detected by fluorescence resonance energy transfer (FRET) [28]. Using human embryonic kidney cells (HEK-293), that were co-transfected with cyan fluorescent protein-fused Cldn1 and yellow fluorescent protein-fused CD81, we could show that FRET efficiency was significantly decreased after treatment with 10 µg/mL cCPE-SSS compared to the untreated control (Figure S1), indicating a reduced Cldn1-CD81 interaction. These results suggested that cCPE-SSS indeed interferes with Cldn1-CD81 co-receptor formation.

### 2.2. S305P/S307R/S313H Substitution in cCPE Improves Its Binding to Cldn1 of Huh7 Hepatoma Cells

**Figure 2:**
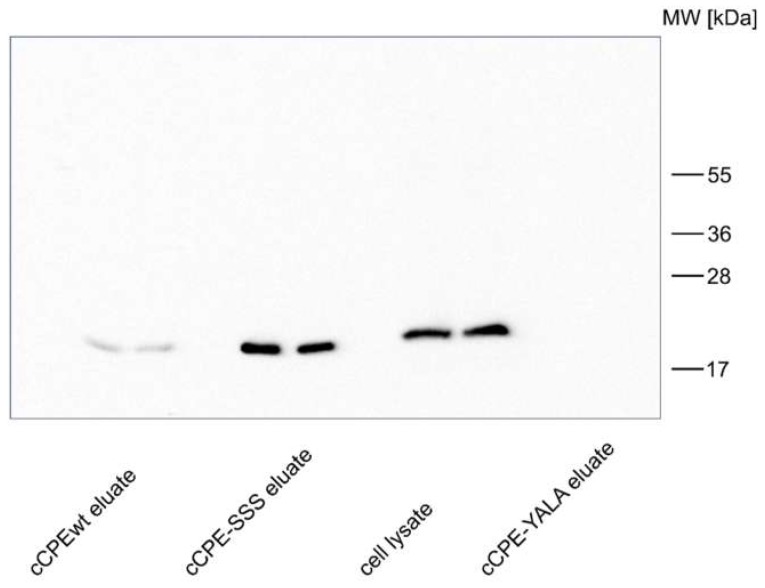
Compared to cCPEwt, cCPE-SSS interacts more strongly with Cldn1 expressed in Huh7 cells. Pull down assays using Huh7 lysates and GST-cCPEwt, -cCPE-SSS or -cCPE-YALA as negative control. Eluates and cell lysate were analyzed in duplicate by SDS-PAGE and Western blot using anti-Cldn1 antibodies. Strong immunoreactive bands in the expected range of ~ 20 kDa were detected for pull down with cCPE-SSS, whereas for cCPEwt, only weak bands and for cCPE-YALA, no bands were detected. Representative results are shown.

The cCPE-variant cCPE-S305P/S307R/S313H (cCPE-SSS) binds more strongly to Cldn1 than cCPEwt [5,6]. To confirm this interaction in a hepatocyte-derived cell culture model, we first used human hepatocarcinoma Huh7 cells [23]. Binding of cCPE variants to Cldn1 endogenously expressed in Huh7 cells was analyzed by means of a pull down assay using GST-cCPE and cell lysates [7]. cCPEwt bound weakly yet specifically to Cldn1 whereas negative control cCPE-Y306A/L315A (cCPE-YALA), which does not interact with claudins [6,45], showed no binding (Figure 2). In contrast, cCPE-SSS showed strong binding to Cldn1 (Figure 2). These data demonstrate that cCPE-SSS binds efficiently to Cldn1 endogenously expressed in hepatocyte model cells.

### 2.3. cCPE-S305P/S307R/S313H Specifically Inhibits HCV infection Of Huh7.5 Cells in a Dose-dependent Manner

**Figure 3:**
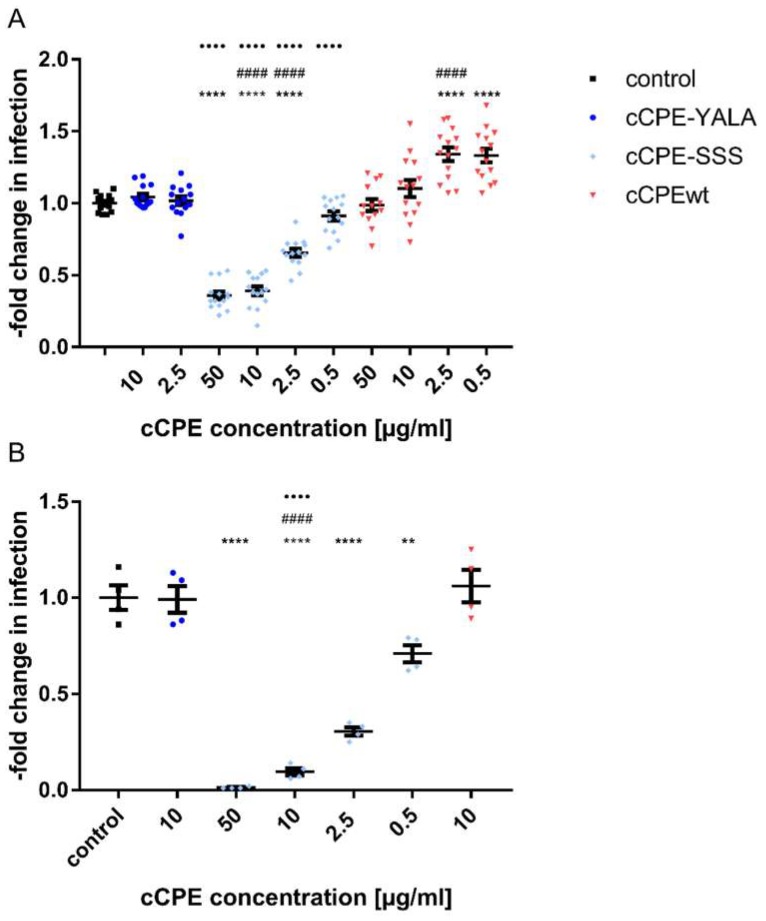
HCVcc infection of Huh7.5 cells in the presence of cCPE variants. Huh7.5 cells were pre-incubated for 2 h at 37 °C with cCPEwt, cCPE-SSS, cCPE–YALA (negative binding control) or not treated prior to infection (control). (**A**) HCVcc was added for 2 h, the medium was changed and 24 h later, HCV infection was analyzed by quantification of infection-positive cells after staining with an anti HCV NS5A antibody. Results are expressed as a percentage of infected cells compared to the control. Results of four experiments with total *n* = 14, for wt 50 *n* = 13. Results shown as means ± SEM. (**B**) HCVcc was added for 26 h. After this, HCV infection was analyzed by quantification of infection-positive cells after staining with an anti HCV NS5A antibody. Results are expressed as a percentage of infected cells compared to control. *n* = 4 Results shown as means ± SEM. One-way ANOVA with post-hoc Sidak’s test was performed to analyze differences between treatment groups. ** *p* ≤ 0.01 vs. control; **** *p* ≤ 0.0001 vs. control; #### *p* ≤ 0.0001 vs. cCPE-YALA; ●●●● *p* < 0,0001 vs. cCPEwt. cCPE-SSS strongly and specifically inhibits Cldn1-mediated HCV infection of Huh7.5 cells in a dose-dependent manner.

Next, we tested whether cCPE-SSS is able to block Cldn1 as a HCV co-receptor and thus to inhibit HCV infection. To this end, infection of human hepatocarcinoma Huh7.5 cells with cell culture-derived HCV particles (HCVcc) was used as an established in vitro HCV infection model [46]. Compared to the parental cell line Huh7, the clone Huh7.5 is more permissive to HCV replication [47]. The infection assay was performed in the absence (control) or presence of cCPE variants. Strikingly, cCPE-SSS inhibited HCV infection of Huh7.5 cells in a dose-dependent manner (0.5–50 µg/mL), whereas the non-binding negative control cCPE-YALA had no inhibitory effect (Figure 3A). Surprisingly, CPEwt significantly enhanced HCV infection at low concentrations (0.5 and 2.5 µg/mL). Increased concentrations of cCPEwt (10 and 50 µg/mL) abolished this enhancing effect but never had an inhibiting effect (Figure 3A).

The inhibitory effect of cCPE-SSS on HCV infection was even more pronounced after extension of HCV incubation of the cells from 2 h to 26 h. Under these conditions, 10 µg/mL cCPE-SSS reduced the amount of HCV-infected cells to 0.095 ± 0.016-fold of the cells which were infected in the non-treated control. In contrast, at this concentration, neither cCPEwt nor cCPE-YALA had a significant effect (Figure 3B). Furthermore, 50 µg/mL cCPE-SSS inhibited the infection of HCV almost completely (0.013 ± 0.002-fold compared to the untreated control).

To exclude that these effects were caused by a reduced cell viability, Huh7.5 cells were treated with different concentrations of cCPEwt or –SSS and cell viability was analyzed with a tetrazolium salt-based colorimetric assay (MTS assay). Neither cCPEwt nor –SSS had adverse effects on Huh7.5 cell viability (Figure S2). This is in accordance with similar results previously obtained with claudin-transfected HEK-293 cells and brain endothelial cells, which were treated with different cCPE variants [8].

Together, the data demonstrate that cCPE-SSS specifically inhibits infection of Huh7.5 cells with HCV.

### 2.4. Both cCPE-SSS and cCPEwt Bind to Huh7.5 Cells

**Figure 4:**
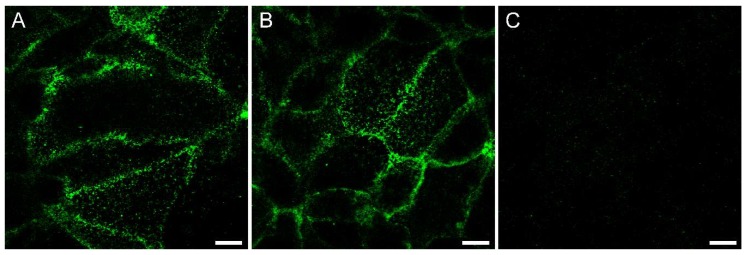
cCPEwt and cCPE-SSS but not cCPE-YALA bind to the surface of Huh7.5 cells. Immunostaining of Huh7.5 cells after 30 min of incubation with cCPE-constructs (10 µg/mL). Detection of GST-cCPEwt (**A**), GST-cCPE-SSS (**B**) and GST-cCPE-YALA (**C**) with anti-GST antibodies. Scale bar 10 µm.

To further investigate the differential effects on HCV infection caused by cCPEwt and cCPE-SSS, the capability of cCPE variants to bind to Huh7.5 cells was analyzed. Confocal microscopy after immunostaining revealed binding of cCPEwt and cCPE-SSS but not of the cCPE-YALA control on the surface of Huh7.5 cells (Figure 4).

**Figure 5:**
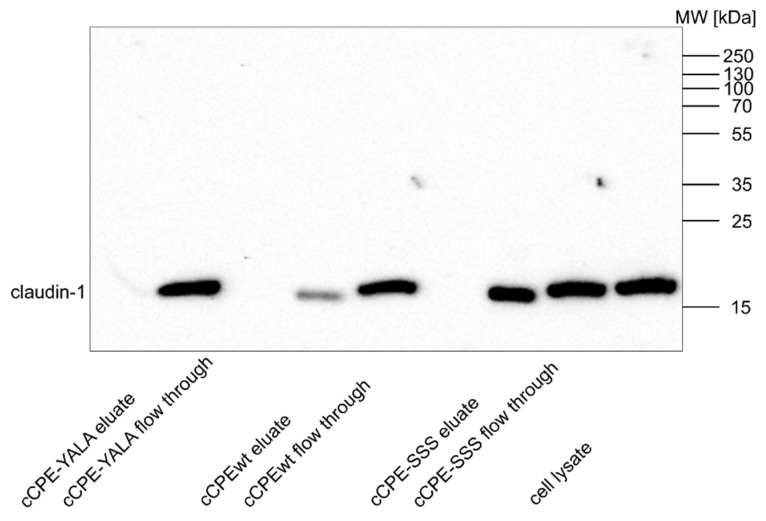
cCPE variants bind to Cldn1 of Huh7.5 cells. Pull down assays using GST-cCPE variants and lysates of Huh7.5 cells were performed. Eluate and flow through were separated by SDS-PAGE and analyzed by Western blot. A strong anti-Cldn1 immunoreactive band in the expected range of ~20 kDa was detected in the pull-down eluates for cCPE-SSS, whereas for cCPEwt, only a weak band and for cCPE-YALA, no band was detected. Representative results are shown.

cCPEwt and cCPE–SSS differ in their affinity towards Cldn1. Their similar binding to Huh7.5 cells indicated that, in addition to Cldn1, other CPE-receptor claudins are expressed in Huh7.5 cells. Expression of Cldn1, -2, and -4, but not of Cldn3, -6, -7, or -9 detected by Western blotting was reported [24]. Since Cldn4 but not Cldn2 is a high affinity CPE-receptor, Cldn4 was a candidate to contribute strongly to the binding of the cCPE variants to Huh7.5 cells. To confirm the presence of cCPE-binding claudins in these cells, pull down assay was performed using the GST-cCPE variants and lysates of Huh7.5 cells and analyzed by Western blot (Figure 5). Cldn1 was clearly detectable in the Huh7.5 lysates. cCPE-SSS interacted more strongly than cCPEwt with Cldn1 whereas no binding was detected with the cCPE-YALA negative control (Figure 5). Hence, concerning the cCPE-Cldn1 interaction with Huh7.5 cells similar results were obtained as for Huh7 cells (Figure 2). However, for Cldn4 no signal was obtained by Western blot neither in the cell lysate nor in the cCPE-bound fraction; for other claudins, also no clear and consistent signals were obtained by Western blot (data not shown).

### 2.5. Huh7.5 Cells Express Cldn1 and Cldn6 as Receptors for cCPE Variants

**Figure 6:**
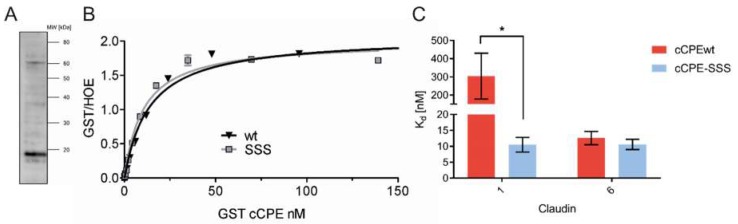
(**A**) Detection of Cldn6 expression in Huh7.5 cells by Western Blot. Huh7.5 cell pellets were lysed, the membrane protein fraction extracted, separated by SDS-PAGE and analyzed by Western blot using anti-Cldn6 antibodies. Cldn6 immunoreactive bands were observed in the expected range (~18 kDa). (**B**) cCPEwt and cCPE–SSS bind with similar affinities to Cldn6 on the cell surface. Binding of GST-cCPEwt and –cCPE-SSS to Cldn6. HEK-293 cells were transfected with Cldn6. Binding of GST-cCPE variants was analyzed using a cellular binding assay with varying cCPE concentrations, fixation, staining with phycoerythrin-coupled anti-GST antibody and Hoechst 33342 (nuclei) for normalization. (**C**) cCPE-SSS binds with similar high affinities to Cldn1 and -6, whereas cCPEwt has a very low affinity for Cldn1. The derived respective affinities of cCPEwt and cCPE–SSS for Cldn6 are not significantly different, but cCPE-SSS binds with a significantly stronger affinity to Cldn1 than cCPEwt does. Dissociation constants (K_d_) were calculated from the binding assays using a non-linear regression analysis in GraphPad Prism 6 (model: Saturation binding, equation: one site—specific binding) [6]. Results are shown as means ± SEM. Student’s t-Test, data from 3 (Cldn1) and 5 to 6 (Cldn6) independent K_d_ calculation experiments, *, *p* < 0.05.

To overcome the limitations connected to sensitivity, specificity and cross-reactivity of anti-claudin antibodies, mass spectrometry was used to identify claudins which are expressed in Huh7.5 cells and able to bind to cCPEwt. A pull-down assay was performed with GST-cCPEwt and lysates of Huh7.5 cells, the cCPEwt-bound fraction as well as the non-bound fraction separated by SDS-PAGE, gel slices corresponding to areas between 15–25 kDa cut and processed for mass spectrometry. Consistent with the Western blots, Cldn1 was detected in the cCPEwt-bound and in the non-bound fraction (Table 1). In contrast, Cldn4 was not found in either fraction. However, Cldn6 was detected in the cCPE-bound fraction (Table 1). Subsequently, literature search-based purchase of another anti-Cldn6 antibody enabled confirmation of Cldn6 expression also by Western blot (Figure 6A). Cldn6 is known to be a CPE-receptor [6,10,48] and a weak entry factor for HCV [23,24]. Expression of Cldn6 mRNA in Huh7.5 cells was previously reported [29]. However, reports on Cldn6 protein expression were not completely consistent. Here, we clearly verify the expression of Cldn6 protein in Huh7.5 cells. Hence, cCPEwt can use Cldn6 and Cldn1 as receptors in Huh7.5 cells. We cannot entirely exclude that other claudins were not detected by mass spectrometry, due to methodological reasons, e.g., unsuitable peptide fragments. However, using mass spectrometry we were previously able to detect claudins such as Cldn1,-3,-4,-7 in liver cells [49].

cCPEwt binds much more strongly to Cldn6 than to Cldn1 [6,9,10]. cCPE-SSS has a much higher affinity for Cldn1 than cCPEwt (Figure 6C) [5,6]. In contrast to cCPEwt, cCPE-SSS binds with high affinity (K_d_~10 nM) both Cldn1 and to Cldn6 (Figure 6B,C). Taken together, the data indicate that high affinity cCPE-SSS binding to Cldn1 is efficiently inhibiting HCV infection of Huh7.5 cells whereas high affinity cCPEwt binding to Cldn6 is not sufficient to inhibit the infection efficiently.

### 2.6. Treatment of Reconstructed Human Epidermis with cCPE Variants Leads to Barrier Opening

To test claudin modulation by cCPE variants in another Cldn1-expressing tissue we analyzed TJ-modulation by cCPE variants in a human in vitro skin model. RHE expressing Cldn1 and Cldn4 [50] was treated with 50 µg/mL of the cCPE variants for 48 h at two different time points (day 4 and day 8). Subsequently, the barrier to ions (trans-epithelial resistance, TER) and the permeability for the tracer molecule Lucifer Yellow (LY) were measured to analyze the barrier function of the RHE.

**Figure 7:**
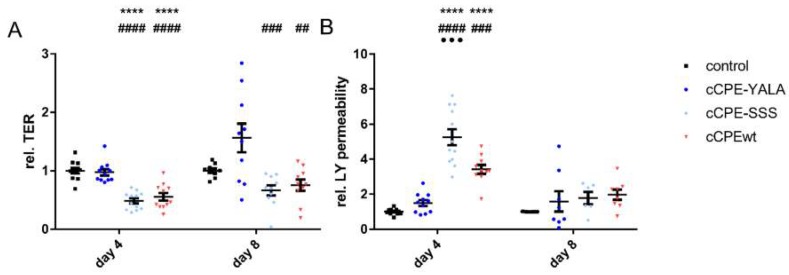
Effect of cCPE variants on (**A**) TER and (**B**) Lucifer Yellow permeability in reconstructed human epidermis. Average relative trans-epithelial resistance (**A**) and relative LY permeability (**B**) after 48 h incubation with 50 µg/mL of either cCPEwt, cCPE-SSS, cCPE-YALA or untreated control on days 4 and 8 after a change to ALI. Results were standardized to respective untreated control. Results are shown as means ± SEM. Statistical significances of one-way ANOVA: * compared to control, # compared to cCPE-YALA, ● compared to cCPEwt. ## *p* < 0.01; ###, ●●● *p* < 0.001; ****, #### *p* < 0.0001. *n* = 5–12.

The treatment with cCPEwt (binding strongly to Cldn4) led to a significant decrease in TER to 0.55 ± 0.07-fold (*p* < 0.0001 compared to untreated RHE and cCPE-YALA, Figure 7A) and a significant increase in LY permeability to 3.42 ± 0.24-fold (*p* < 0.0001 compared to the negative control and *p* < 0.001 compared to cCPE-YALA) on day four. However, on day eight, only a slight reduction in TER (0.753 ± 0.09-fold, Figure 7A, *p* < 0.01 compared to to cCPE-YALA) and a slight increase in LY permeability (1.97 ± 0.27-fold), could be observed (Figure 7B).

On day four, compared to cCPEwt, cCPE-SSS (binding strongly to Cldn1 and Cldn4) had a similar effect on TER (0.49 ± 0.04-fold, *p* < 0.0001 compared to untreated control and cCPE-YALA, Figure 7A) and a significantly greater effect on LY permeability (5.23 ± 0.44-fold, *p* < 0.0001 compared to untreated control and cCPE-YALA, *p* < 0.001 compared to cCPEwt, Figure 7B). On day eight, TER was significantly decreased after treatment with cCPE-SSS (0.66 ± 0.08-fold, *p* = 0.001 compared to untreated control, Figure 7A) and there was a slight tendency for an increased outside-to-inside passage of LY (1.78 ± 0.32%, Figure 7B) for cCPE-SSS.

The different cCPE variants had no effect on the expression of the relevant claudins in the RHE, (Cldn1, -3, -4 and -7), on day 4 or on day 8 (data not shown).

In summary, the data showed that cCPE variants can be used as TJ modulators in the skin and that removal of Cldn1 and Cldn4 from the TJ impairs the barrier properties of the human epidermis.

## 3. Discussion

In this study, we showed that Cldn1 expressed in liver and skin can be efficiently targeted with the broad-specificity claudin binder cCPE-SSS. In contrast to cCPEwt, cCPE-SSS strongly inhibited Cldn1-dependent HCV infection in a hepatocyte in vitro model. In addition, cCPE-SSS improved paracellular permeability for small molecules in an in vitro human skin model to a larger extend than cCPEwt.

Claudins have been suggested as targets for diverse therapeutic application throughout numerous studies, including targeting of claudin-overexpressing cancer entities [51,52], visualization of upregulated claudin expression in colon polyps [53], opening of tissue barriers, e.g., the blood-brain-barrier [8] or intestinal barriers [54,55].

Use of monoclonal antibodies for claudin targeting led to very promising in vivo results; in particular with anti-Cldn18 or -Cldn1 antibodies for treatment of gastric cancer or HCV infections, respectively [14,18,32,33,56,57]. As an alternative for claudin targeting, application of CPE or cCPE variants has been suggested [8,58,59,60]. Not only is the bacterial production of these proteins very cost-effective compared to antibody production, but cCPE can also be adjusted to target several claudin subtypes. The latter is advantageous e.g., in viral infections like hepatitis C, where a pan-genomic treatment and prevention of resistance toward treatment (e.g., due to viral use of multiple claudins as receptors) is desirable. However, as claudins are expressed throughout the entire body, a thorough evaluation of possible adverse effects of claudin-targeting agents is necessary. Full-length CPE is known to have a high systemic toxicity. In contrast, more than ten weekly intravenous administration of 5 mg/kg cCPE did not increase biochemical markers of kidney and liver injury in mice [61]. These findings suggest that cCPE variants have limited and controllable adverse side effects even when applied systemically. Nevertheless, in vivo effects on tissue barriers have to be investigated in more detail. At least, blocking of extra-junctional HCV co-receptors might be achieved at much milder cCPE doses than opening of preformed junctional barrier, as indicated by the data (Figure 3, Figure 7).

Host-targeting agents are promising therapeutic approaches for novel anti-viral treatments for patients that do not respond to conventional therapies of direct-acting antivirals, or combination of ribavirin and pegylated interferon. So far, antibodies recognizing the extracellular domains of various cellular HCV receptors, for example Cldn1 [14,32,33], CD81 [62] and SR-BI [63] have been successfully used in vitro and in vivo to prevent HCV infection.

We showed that the broad-specificity claudin binder cCPE-SSS can effectively inhibit HCV infection in the hepatoma cell line Huh7.5. The substitution S305P/S307R/S313H improved cCPE binding to Cldn1 of Huh7 cells significantly compared to cCPEwt, providing a tool to target hepatoma cells potently. In contrast to cCPEwt, cCPE-SSS inhibited HCVcc infection of hepatoma Huh7.5 cells in a dose-dependent manner (Figure 3). Surprisingly, low concentrations of cCPEwt even aggravated HCV infection. Due to its much lower affinity to Cldn1 than to Cldn6, cCPEwt should bind mainly to Cldn6 under these conditions. We assume that Cldn1 and Cldn6 compete with each other for CD81 association, that cCPEwt binding to Cldn6 interferes with Cldn6-CD81 interaction (similar as for cCPE-SSS – Cldn1, indicated by homology modeling) and that in turn formation of the more potent Cldn1-CD81 HCV co-receptor complex is favored. Such a competition mechanism might explain aggravation of infection by cCPEwt. 

Cldn1-specific neutralizing antibodies only partially inhibited infection of Huh7.5 cells by HCV strains with broad claudin tropisms, suggesting a possible escape from Cldn1-specific targeting molecules through Cldn6 or Cldn9 in cell culture [64]. In addition, a previously Cldn1-dependent HCV strain was shown to evolve the capacity to use Cldn6 as a host entry factor via a single amino acid mutation in the HCV E1 protein [65]. cCPE-SSS concomitantly targets Cldn1 and Cldn6 on the surface of Huh7.5 with nanomolar affinities (Figure 5 and Figure 6). Hence, use of cCPE-SSS as a broad spectrum claudin binder might avoid the escape from Cldn1-specific antibodies, making it a more powerful agent in HCV treatment.

In summary, we showed that cCPE can be modified and used to prevent infection of hepatoma cells with HCV by binding to claudins including Cldn1, thus inhibiting formation of the viral entry complex.

Additionally, we analyzed the potential of cCPE variants to modulate the epidermal barrier by targeting claudins of the SG in RHE models. A purposeful and directed opening of the epidermal barrier can be useful to improve transdermal drug delivery [61,66].

Transdermal drug delivery is a desirable method of application. Its numerous benefits include a non-invasive technique, avoidance of first-pass metabolism by digestive enzymes and thus a better bioavailability of poorly orally bioavailable drugs, a constant plasma drug concentration as well as enhancement of patient compliance [61,66]. Treatment of RHE with cCPE variants prevents consecutive incorporation of claudins in TJs and allowed us to study the effects of TJ rearrangements on the epidermal barrier.

cCPEwt removes Cldn3, -4, -6, -7, -8 and -14 from the TJ. However, its affinity for Cldn1 is very low. We show here that cCPEwt resulted in an impaired epidermal barrier to small molecule tracers (LY) in the RHE at day 4, i.e., during formation of the SC. Impairment of the epidermal barrier to molecular tracers after cCPEwt treatment during formation of SC has been described earlier [39], and is in accordance with our results. In the study by Yuki et al., 10 or 50 µg/mL cCPEwt was added to the culture medium at the beginning of SC formation. After a four-day incubation period, the human skin equivalent models showed a significant increase in LY permeability, indicating a weakened epidermal barrier. [39]. In addition, we demonstrate that the ion barrier was weakened by cCPEwt on day 4. On day 8, when SC was more mature, cCPEwt did not have a significant effect even though there was a trend.

cCPE-SSS has a higher affinity for Cldn1-9 [5,6], which should result in a greater barrier impairment compared to cCPEwt because Cldn-1 plays a major role in epidermal barrier function [35]. Indeed, in the four-day model, the cCPE-SSS treatment led to a significant decrease in TER and a significant increase in LY permeability. This effect was greater than the effect of cCPEwt.

In the eight-day model, the TER was significantly lower after cCPE-SSS treatment compared to the other groups. This indicates that loss of Cldn1 from the TJ weakens the ionic barrier even in a well-developed SC.

All observed effects were weaker in the eight-day compared to the four-day model although the cCPE variants were applied from the basolateral side and thus were not held up by the SC. This finding might indicate a reduced accessibility of claudins in the more mature epithelium. If the extracellular domains of claudins are involved in trans-interactions, cCPE cannot bind anymore [67]. Likewise, the contribution of the TJ to epidermal barrier sealing might be less strong in the presence of a mature and intact SC.

The most effective approach to improve transdermal drug delivery should modify both epidermal barriers, the SC as well as the TJ. Hence, cCPE-based biologics might be used in combination with a chemical absorption enhancer, like surfactants (surface-active agents), fatty-acid esters, terpenes and solvents. Chemical enhancers increase skin permeability by various means, including solubilization of lipids and denaturing of keratin as well as fluidizing the crystalline structure of the SC [68]. Combining these methods with the directed claudin-targeting by cCPE could have a therapeutically significant effect on the epidermal barrier.

In conclusion, cCPE variants are promising tools for claudin targeting in diverse tissues including liver and skin. Their claudin binding properties can be adjusted to fit a certain demand, their production is very cost-effective compared to monoclonal antibodies and their systemic administration so far has been without fatal adverse side effects.

## 4. Materials and Methods

### 4.1. Structural Bioinformatics and Molecular Modeling

Homology model of the Cldn1-cCPE-SSS complex was built based on the Cldn4-cCPE complex (PDB ID: 5B2G; [43]). All manipulations, optimizations of the models, as well as calculations of hydrophobic and electrostatic potentials on the molecular surfaces, were performed with Sybyl X2.1.1 (Certara USA Inc., St. Louis, MO, USA). Models were energetically minimized using the AMBER7 FF99 force field. Structure images were generated with PyMOL Molecular Graphics System 1.8.4.1 (Schrödinger LLC, 2017).

### 4.2. Chemicals and Solutions

Antibodies: Phycolink^®^ anti-GST R-phycoerythrin-conjugated antibodies (Europa Bioproducts Ltd., Cambridge, UK), rabbit anti-Cldn1 and anti-Cldn4, (Invitrogen, Carlsbad, USA), rabbit polyclonal anti-Cldn6 (Immuno-Biological Laboratories, Fujioka, JP), mouse anti-GST (Sigma-Aldrich, Steinheim, DE), mouse anti-NS5a (ABCAM, Cambridge, UK).

Chemicals: Lucifer Yellow: Sigma-Aldrich Chemie GmbH (München, DE).

### 4.3. Cells and Tissues

#### 4.3.1. Cultivation of Cells

Primary human keratinocytes were isolated from juvenile foreskin as described previously [50]. The use of these samples was in accordance with approval of the ethics committee of the Ärztekammer Hamburg (WF 61/12). Samples were used anonymously and all investigations were conducted in accordance to the principles of the Declaration of Helsinki.

Human Huh7 cells [23] kindly provided by Dr. Nora Gehne (FMP), were cultured in Dulbecco’s modified Eagle’s medium supplemented with 10% (v/v) FBS and 1% (v/v) penicillin/streptomycin solution (10,000 U/mL penicillin, 10,000 µg/mL streptomycin, Gibco™, Thermo Fisher Scientific, Waltham, MA, USA). Huh7.5. cells (Apath, LLC, St. Louis, MO, USA) were cultured as described previously [69].

HEK-293 cells (CRL-1573, A.T.C.C., Manassas, VA, USA), [70] were kindly provided by Professor Otmar Huber (Friedrich Schiller University of Jena), cultivated in Minimal Essentail Medium with Earle’s salts and L-glutamine (Merck KGaA, Darmstadt, DE) supplemented with 10% (v/v) FBS and 1% (v/v) penicillin/streptomycin solution (10000 U/mL penicillin, 10,000 µg/mL streptomycin, Gibco™, Thermo Fisher Scientific, Waltham, MA, USA). For transfection, cells were seeded onto coverslips with a diameter of 32 mm (Menzel Gläser, Thermo Fisher Scientific, Waltham, MA, USA). At 80% confluency, cells were transfected with 2.9 ng plasmid encoding N-terminally YFP-fused human CD81 and 1.1 ng plasmid encoding N-terminally CFP-fused Cldn1 using polyethlenimine (PEI MAX 40K, Polysciences, Inc., Warrington, PA, USA). Cells were further incubated at 37 °C for 2 days before measuring FRET efficiency. In the case of cCPE-SSS treatment, cells were incubated with 10 µg/mL of the protein for 30 min prior to starting the measurement.

#### 4.3.2. HCVcc Infection Assay

Two to three days after plating on 48 well plates, Huh7.5 cells were incubated for 2 h with GST- cCPEwt, GST-cCPE-SSS or GST-cCPE-YALA at concentrations between 0.5–50 µg/mL at 37 °C or not further treated (control). After incubation the cells were infected with Gt2a HCV J6CF-JFH1 (Jc1) at a multiplicity of infection (moi) of 1.0 at 37 °C. The medium was changed 2 h after infection and Huh7.5 cells were incubated at 37 °C for further 24 h or Huh7.5 cells were incubated at 37 °C for 26 h without medium exchange.

Huh7.5 cells were fixed with acetone/methanol (1:1 ratio), incubated for 1 h at room temperature with anti NS5a antibodies, and incubated for one hour at room temp with Alexa Fluor 488-conjugated goat anti-mouse secondary antibody (washed with PBS between steps) and counterstained with 4′,6-diamidino-2-phenylindole (DAPI). Infection levels were determined using a Nikon Eclipse TE2000-U microscope (Nikon Corporation, Tokyo, Japan), and expressed as a fold change of the cCPE untreated control [46,69].

#### 4.3.3. MTS Cell Proliferation Assay

Cellular toxicity was measured using a Cell Titer 96 aqueous nonradioactive cell proliferation assay kit, according to the manufacturer’s instructions (Promega, Madison, WI, USA) as described previously [71]. Briefly, 2 × 10^4^ Huh7.5 cells were seeded in a 96 well plate. After cultivation for 72 h, cells were incubated with cCPEwt and cCPE-SSS at concentrations ranging from 0 to 100 ng/µL for 2 h. 3-(4,5-dimethylthiazol-2-yl)-5-(3-carboxymethoxyphenyl)-2-(4-sulfophenyl)-2*H*-tetrazolium) (MTS)-phenazine methosulfate solution (40 μL) was added to each well and the samples were incubated at 37 °C in a humidified 5% CO_2_ atmosphere for 3 h. Absorbance was measured at 490 nm with a UVmax plate reader (Molecular Devices, San José, CA, USA). Each sample was analyzed in triplicate.

#### 4.3.4. Live Cell Imaging, Acceptor Photobleaching and FRET Data Analysis

Live cell imaging and photo bleaching were performed using the laser scanning microscope LSM 780 (Carl Zeiss Microscopy GmbH, Jena, DE). Experiments were performed as described previously [72].

FRET efficiency as calculated using the following formula: E_FRET_ = (CFP_A_ – CFP_B_)/CFP_A_; CFP_A_ and CFP_B_ referring to CFP fluorescence intensity after and before photobleaching, respectively.

#### 4.3.5. Cultivation of Keratinocytes and Creation of Reconstructed Human Epidermis

Reconstructed human epidermis was generated from primary human keratinocytes as already described [50]. Briefly, the 3 × 10^5^ cells were seeded in 500 µL EpiLife medium with 1.5 mM CaCl_2_ onto Millicell cell culture inserts (Merck Millipore, Tullagreen, Ireland) and 2.5 mL cell culture medium was applied to the basal compartment. The cells were cultivated at 37 °C in humidified atmosphere for 30 h before lifting to the air-liquid interface (ALI). Concurrently, the basal medium was exchanged with 1.5 mL of EpiLife medium containing 1.5 mM CaCl_2_, 92 µg/mL ascorbic acid (Sigma, Darmstadt, DE) and 10 ng/mL recombinant human keratinocyte growth factor (R&D Systems, Wiesbaden-Nordenstadt, DE). Cells derived from an individual donor were cultured as duplicates. 

#### 4.3.6. Treatment of Reconstructed Human Epidermis with cCPE and Barrier Assays

For cCPE treatment, 50 µg/mL of the respective cCPE mutants were added to the basal compartment of the RHE models at day 2 or 6 after transitioning to ALI. After 48 h of incubation at 37 °C in a humidified atmosphere, the transepidermal resistance as a measure for the epidermal ion barrier was measured using chopstick electrodes. Relative TER values were used due to intrinsic variation in starting absolute TER values between the different RHE cultures since they were derived from different human donors. The absolute TER values of the control RHE cultures were between 0.53 and 2.26 kOhm × cm^2^ at day 4 and 0.71 and 2.92 kOhm × cm^2^ at day 8.

To examine the outside-to-inside epidermal barrier for small molecules, the RHE models were transferred to 6-well plates with 1.5 mL of medium. Then, 200 µl of a 1 mM Lucifer Yellow solution was added to the apical side. After 6 h incubation at 37 °C, 100 µl of the basal medium was transferred into a microtiter plate. The LY fluorescence was measured with the Infinite M2000 fluorescence reader (Tecan Group Ltd., Männedorf, Switzerland) using the following settings: excitation wavelength 425 nm, emissions wavelength 550 nm, 10 flashes, 50-fold gain. By measuring the fluorescence of LY standard samples of known concentration, the concentration of the basal media samples could be calculated. With this, the LY permeability was calculated using the following formula: $P_{APP}\;\left[\frac{cm}{s}\right]=\frac{flux\;\left[\frac{µg}{s\;\cdot \;cm^{2}}\right]}{LY\;concentration\;\left[\frac{µg}{ml}\right]}$.

### 4.4. Plasmids

Plasmid encoding GST-CPE194-319wt (GST-cCPE), GST-cCPE-S305P/S307R/S313H and GST-cCPE–Y306A/L315A fusion protein were reported previously [6], as well as plasmid encoding N-terminally CFP-tagged Cldn1 [72]. pEZ-M15-NYFP/huCD81 was obtained from GeneCopoeia™ (Rockville, MD, USA).

### 4.5. Expression and Purification of cCPE-Constructs

CPE194-319 (cCPE) with N-terminal GST-fusions as well as GST (control) were expressed in *E. coli* BL21 as reported previously [6]. Briefly, bacteria were grown to A_600_ = 0.6–0.8, expression induced by addition of 1 mM isopropyl-β-D-thiogalactopyranoside. 3 h after induction, bacteria were harvested (10 min, 20,000× *g*, 4 °C) and lysed in PBS (phosphate buffered saline) with 1% (v/v) Triton X-100, 0.1 mM PMSF, 1 mM EDTA, protease inhibitor cocktail (Sigma-Aldrich, Steinheim, DE) and sonicated with Vibra Cell™ Model 72434 (BioBlock Scientific, Strasbourg, France) by 10 × 1 s pulses. Insoluble cell debris was removed by centrifugation (20,000× *g* for 1 h at 4 °C). Proteins were purified from supernatants using glutathione-agarose (Sigma-Aldrich, Steinheim, DE) and dialyzed against PBS. Protein concentration was determined with BCA Protein Kit (Thermo-Scientific, Rockford, USA).

### 4.6. Pull-down Assay

Pull-down assay was performed as described previously [10]. Briefly, Huh7 and Huh7.5 cells were lysed with 1% Triton X-100, EDTA-free protease inhibitor cocktail (Roche, Mannheim, DE) in PBS. The 10,000× *g* supernatant was incubated with GST-cCPE constructs bound to Glutathione Sepharose beads (GE Healthcare, Uppsala, Sweden) for 2 h on a shaker at 4 °C. Beads were washed 3 times with PBS containing 0.5% Triton X-100, bound proteins eluted with Laemmli buffer and analyzed by SDS-PAGE and Western blot with anti-Cldn1, or anti-Cldn4 antibodies. After stripping, membranes were incubated with anti-GST antibodies to verify that similar amounts of GST-cCPE were bound to the beads.

### 4.7. Immunostaining

Immunocytochemistry was performed as described [10]. Briefly, 2–3 days after plating, Huh7.5 cells were incubated with 10 µg/mL GST-cCPEwt, GST-cCPE-SSS or GST-cCPE-YALA for 30 min at 37 °C and washed with PBS. Cells were fixed with methanol/acetone 1:1 ratio, blocked with 1% BSA in PBS, incubated with anti-GST antibodies, washed, and incubated with Alexa Fluor 488-conjugated goat anti-mouse secondary antibodies. Cells were analyzed with the LSM 510 META confocal microscope (Zeiss, Jena, DE).

### 4.8. Mass Spectrometry

#### 4.8.1. In-gel Trypsin Digestion

Gel bands of interest were excised after Coomassie blue staining and cut into 1–2 mm^3^ gel pieces, which were placed into 1.5 mL sample tubes. Gel pieces were rinsed twice with wash solution for 18 h in total (200 μL, 50% methanol, 5% acetic acid). The solutions were removed and gel pieces were dehydrated in acetonitrile (200 μL, 5 min). Supernatants were removed and gel pieces were dried in a vacuum centrifuge for 3 min. Disulfide reduction was performed with 10 mM DTT (30 μL) for 0.5 h, followed by alkylation with 100 mM iodoacetamide (30 μL) for 0.5 h. Supernatants were removed from the gel samples and dehydration with acetonitrile and evaporation performed as described above. Gel pieces were washed with 100 mM ammonium bicarbonate (200 μL, 10 min). Supernatants were removed and dehydration performed with acetonitrile and evaporation as above. The gel samples were then rehydrated on ice with freshly prepared trypsin solution (30 μL, 20 ng/μL sequencing grade trypsin (Promega, Madison, WI, USA) in 50 mM ammonium bicarbonate). After rehydration, excess trypsin solution was removed and 50 mM ammonium bicarbonate (10 μL) was added to prevent dehydration of gel pieces. Gel samples were digested at 37 °C for 18 h. The gel pieces were then extracted sequentially with 50 mM ammonium bicarbonate (60 μL), 50% acetonitrile, 5% formic acid (60 μL) and 85% acetonitrile, 5% formic acid (60 μL). The combined extracts were evaporated in a vacuum centrifuge and were re-dissolved in 5% acetonitrile, 0.1% formic acid (20 μL) on an ultrasonic bath and transferred into LC-MS sample vials.

#### 4.8.2. LC-MS/MS Analysis

For the analysis of in-gel digested protein material, liquid chromatography was performed using an Ultimate 3000 nano-HPLC system (Dionex, Sunnyvale, CA, USA) comprising a WPS-3000 micro auto sampler, a FLM-3000 flow manager and column compartment, a UVD-3000 UV detector, an LPG-3600 dual-gradient micro-pump, and an SRD-3600 solvent rack controlled by Hystar (Bruker Daltonics, Billerica, MA, USA) and DCMSLink 2.0 software (Dionex, Sunnyvale, CA, USA). Samples were concentrated on a trapping column Dionex (Sunnyvale, CA, USA), 300 μm i.d., 0.1 cm) at a flow rate of 20 μL/min. For the separation with a C18 Pepmap column (75 μm i.d., 15 cm, Dionex), a flow rate of 250 nL/min was used as generated by a cap-flow splitter cartridge (1/1000). Peptides were eluted by the application of a 30 min multi-step gradient using solvents A (98% H_2_O, 2% acetonitrile, 0.1% formic acid) and B (80% acetonitrile, 20% H_2_O, 0.1% formic acid) (Table 2):

The liquid chromatography was interfaced directly with a 3D high capacity ion trap mass spectrometer (amaZon; Bruker Daltonics, Billerica, MA, USA) utilizing 10 μm i.d. distal coated SilicaTips (New Objective, Woburn, MA, USA) and nano-ESI mode. SPS parameter settings on the ion trap were tuned for a target mass of 850 *m/z*, compound stability 100% and a smart ICC target of 250,000. MS/MS analysis was initiated on a contact closure signal triggered by HyStar software (version 3.2). Up to five precursor ions were selected per cycle with active exclusion (0.5 min) in collision-induced dissociation (CID) mode. CID fragmentation was achieved using helium gas and a 30–200% collision energy sweep with amplitude 1.0 (ions are ejected from the trap as soon as they fragment).

#### 4.8.3. Data Processing and Database Searching

Raw LC-MS/MS data were processed and Mascot compatible files were created using DataAnalysis 4.0 software (Bruker Daltonics, Billerica, MA, USA). Database searches were performed using the Mascot algorithm (version 2.4) and the UniProt_SwissProt database with mammalian taxonomy restriction (v2012.09.17, number of entries 537,505, after taxonomy filter: 66,032). The following parameters were applied: 2+, 3+ and 4+ ions, peptide mass tolerance 0.3 Da, ^13^C = 2, fragment mass tolerance 0.6 Da, number of missed cleavages: two, instrument type: ESI-TRAP, fixed modifications: Carbamidomethylation (Cys), variable modifications: Oxidation (Met).

### 4.9. Statistical Analysis

Results are expressed as means ± standard error of the mean (SEM). Gaussian distribution was tested using the D´Agostino-Pearson omnibus test. Statistical analyses were performed using one-way ANOVA with post-hoc Sidak’s test or Student´s t-test (in case of failed normality test: Mann-Whitney test), as indicated. *p* < 0.05 was considered statistically significant.

## Figures and Tables

**Table 1:** Mass spectrometry confirmed Cldn1 and Cldn6 protein expression in Huh7.5 cells. Pull down assay with GST-cCPEwt and lysates of Huh7.5 cells. cCPEwt-bound and non-bound fractions were separated by SDS-PAGE; gel slices corresponding to areas between 15–25 kDa were cut and processed for mass spectrometry.

Fraction	Cldn	Mascot Score	Sequence Cover-Age	Peptides	Position	Ions Score	Mass Determined	Mass Expected
E1: ~15kD	6	96	12%	K.VYDSLLALPQDLQAAR.A	66–81	46	1771.8914	1771.9468
				R.DFYNPLVAEAQK.R	146–157	90	1393.6274	1393.6878
E2: ~17KD	1	144	14%	K.VFDSLLNLSSTLQATR.A	66–81	91	1763.9194	1763.9418
				R.IVQEFYDPMTPVNAR.Y	144–158	56	1778.7754	1778.8662
				R.IVQEFYDPMTPVNAR.Y Oxidation M	144–158	80	1794.7974	1794.8611
E2: ~17KD	6	111	17%	K.VYDSLLALPQDLQAAR.A	66–81	77	1771.8914	1771.9468
				R.DFYNPLVAEAQK.R	146–157	71	1393.6614	1393.6878
				R.YSTSAPAISR.G	200–209	43	1051.4934	1051.5298
E3: ~18KD	6	88	17%	K.VYDSLLALPQDLQAAR.A	66–81	57	1771.8774	1771.9468
				R.DFYNPLVAEAQK.R	146–157	66	1393.6734	1393.6878
				R.YSTSAPAISR.G	200–209	32	1051.5194	1051.5298
FT9: ~17KD	1	97	14%	K.VFDSLLNLSSTLQATR.A	66–81	82	1763.9034	1763.9418
				R.IVQEFYDPMTPVNAR.Y	144–158	55	1778.7994	1778.8662

**Table 2:** Elution protocol of peptides. A C18 Pepmap column (75 µm i.d., 15 cm) was used for separation of peptide fragments. Solvent A (98% H_2_O, 2% acetonitrile, 0.1% formic acid) and solvent B (80% acetonitrile, 20% H_2_O, 0.1% formic acid) were used in the ratio indicated.

Composition (% Solvent B)	Run Time (min)
2–10	0–3
10–25	3–18
25–50	18–30
50–90	30–30.2

## Supplementary Materials

Supplementary materials can be found at https://www.mdpi.com/1422-0067/20/19/4774/s1.

## Abbreviations

ALI	air-liquid interface
ANOVA	analysis of variance
CD81	cluster of differentiation 81
CFP	cyan fluorescent protein
Cldn	claudin
cCPE	amino acids 194–319 of *Clostridium perfringens* enterotoxin
cCPE-SSS	cCPE-S305P/S307R/S313H
cCPEwt	wildtype cCPE
cCPE-YALA	cCPE-Y306A/L315A
CPE	*Clostridium perfringens* enterotoxin
ECS	extracellular segment
FBS	fetal bovine serum
FRET	fluorescence resonance energy transfer
GST	Glutathione S-transferase
HCV	hepatitis C virus
HCVcc	cell culture-derived hepatitis C virus particles
LC/MS-MS	liquid chromatography - tandem mass spectrometry
LY	Lucifer yellow
MTS	3-[4,5,dimethylthiazol-2-yl]-5-[3-carboxymethoxy-phenyl]-2-[4-sulfophenyl]-2H -tetrazolium
RHE	reconstructed human epidermis
SC	*stratum corneum*
SEM	standard error of the mean
SG	*stratum granulosum*
SR-BI	scavenger receptor class B type I
TER	trans-epithelial resistance
TJ	tight junction
YFP	yellow fluorescent protein
