# Supplementary material for: Use of Modified Clostridium perfringens Enterotoxin Fragments for Claudin Targeting in Liver and Skin Cells
Source: Int J Mol Sci. 2019 Sep 26;20(19):4774. doi: 10.3390/ijms20194774 (PMC6801472; doi:10.3390/ijms20194774)
Supplement: Supplementary file 1 [file ijms-20-04774-s001.pdf]

International Journal of *Molecular Sciences*

Supplementary Materials:

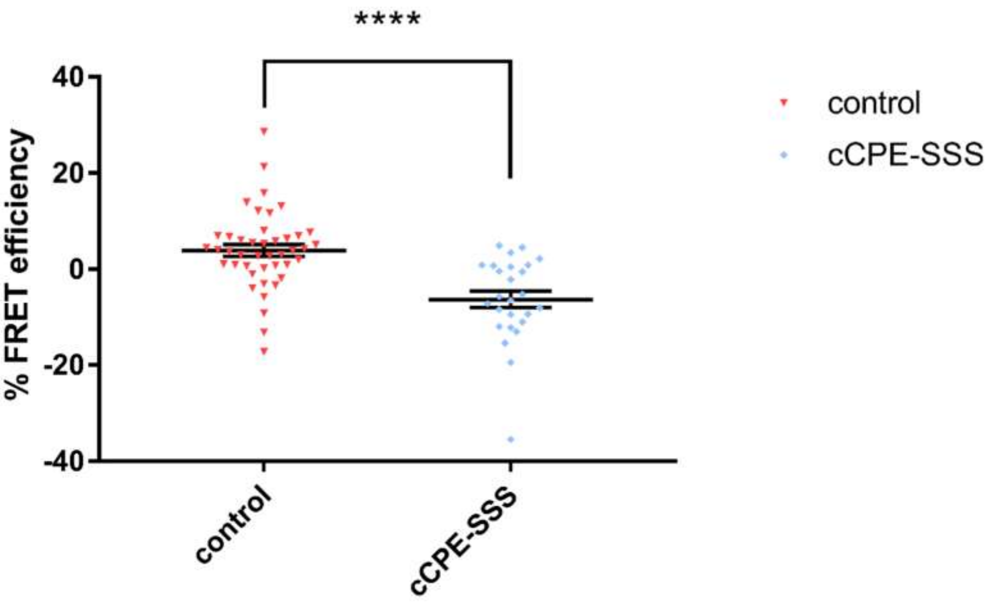

Figure S1 cCPE-SSS interferes with Cldn1-CD81 interaction. HEK-293 cells were co-transfected with cyan fluorescent protein-fused Cldn1 and yellow fluorescent protein-fused CD81. Cells were incubated with 10 µg/ml cCPE-SSS for 30 min at 37 °C prior to FRET measurements. Control was not treated. Cldn1-CD-81 interaction was quantified with the FRET acceptor photo bleaching method. Negative FRET efficiency might result from CFP donor bleaching. Results are shown as means ± SEM. Statistical significance of Mann-Whitney test: **** $p < 0.0001$. control: $n = 43$, cCPE-SSS: $n = 26$, two independent experiments.

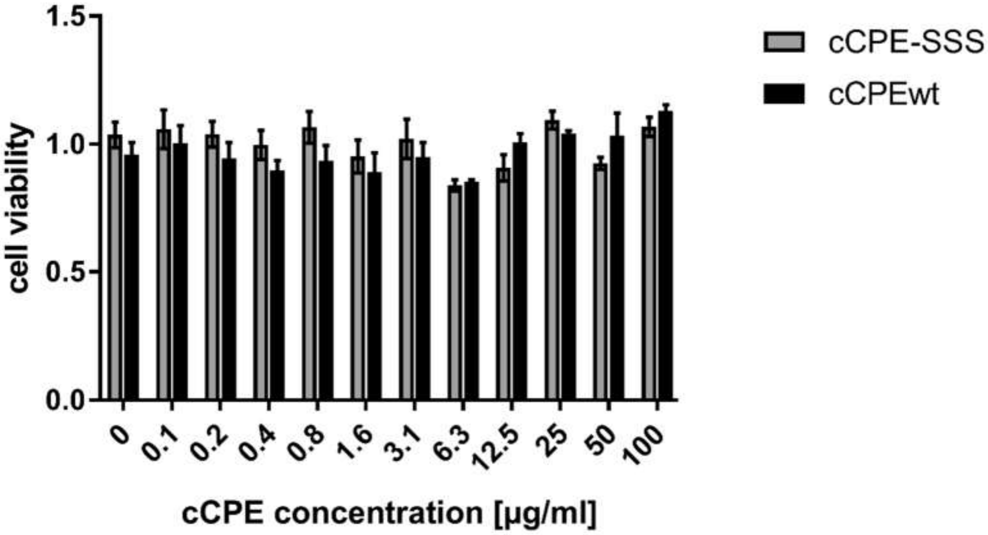

Figure S2 cCPE-SSS treatment has no adverse effect on viability of Huh7.5 cells compared to cCPEwt. Huh7.5 cells were seeded in 96 well plate, cultivated for 72 h and treated with cCPEwt or –SSS for 2 h. MTS reagent was added for 3 h (37 °C, 5% CO_2) and absorbance was measured at 490 nm. Cell viability is directly proportional to absorbance at 490 nm. Student´s t-test showed that the viabilities of the two treatment groups were not significantly different at any concentration. $n = 3$.
